# Performance Analysis of PLA Material Based Micro-Turbines for Low Wind Speed Applications

**DOI:** 10.3390/polym14194180

**Published:** 2022-10-05

**Authors:** Belqasem Aljafari, Devakirubakaran Samithas, Praveen Kumar Balachandran, Sambandam Anandan, Thanikanti Sudhakar Babu

**Affiliations:** 1 Department of Electrical Engineering, College of Engineering, Najran University, Najran 11001, Saudi Arabia; 2 Department of Electrical and Electronics Engineering, QIS College of Engineering and Technology, Ongole 523272, India; 3 Department of Electrical and Electronics Engineering, Vardhaman College of Engineering, Hyderabad 501218, India; 4 Nanomaterials & Solar Energy Conversion Lab, Department of Chemistry, National Institute of Technology, Tiruchirappalli 620015, India; 5 Department of Electrical and Electronics Engineering, Chaitanya Bharathi Institute of Technology (CBIT), Hyderabad 500075, India

**Keywords:** micro turbine, PLA material, wind plant, horizontal axis

## Abstract

Various studies have been conducted in recent years to find solutions to the issues in wind energy conversion systems. A 100W horizontal axis micro wind turbine is built for low wind speed applications in this work. The Blade Element Momentum theory approach was used to design the 100W micro wind turbine blade. The wind turbine blade 3D model was created using the CREO CAD 3.0 software. Based on the aerodynamic studies, the airfoil S9000 is chosen among others for generating high power at low wind speed. The density, Young’s modulus, and the Poisson ratio of the proposed wind turbine blade model with acrylonitrile butadiene styrene (ABS) and polylactic acid (PLA) materials were compared. ABS and PLA materials were investigated using a 0.33 mm layer of infill ranging from 10% to 100%. PLA and ABS output values were compared in terms of deformation, equivalent stress, and equivalent strain. PLA materials, on the other hand, have less deformation and greater structural properties than ABS materials. The wind blade structural analysis was performed in ANSYS 15 software, and the details of experimental and simulated results are presented in this paper.

## 1. Introduction

Natural energy sources can be converted and used for a variety of useful uses. The earth’s accessible energy sources are divided into two categories: conventional and non-conventional sources. Due to the rapid depletion and contamination of conventional sources, the entire world seeks alternatives. Renewable energy system research has received increased attention in recent decades. The use of renewable energy sources has significant challenges, such as environmental change, inconsistent availability, and so on. Nature provides several renewable energy sources, such as solar, wind, and bio-gas. Because of its availability, the wind energy conversion system (WECS) has been developed in recent years. The wind energy conversion system operates by converting the kinetic energy of the wind into electricity [1,2]. Later, power conditioning devices, such as inverters, converters, and filters, process the converted electrical energy. For efficient energy conversion, the wind turbine selection is very crucial. The WECS uses two different types of wind turbines to transform the kinetic energy of the wind into electrical energy: the horizontal axis wind turbine (HAWT) and the vertical axis wind turbine (VAWT). The power conversion rate can be improved by using the right wind blade structure, turbine material, and structural analysis. The chord, twist, solidity, angle of attack, and pitch angle of the wind turbine blade must all be constructed with a high lift force and low drag force. The cited articles are used to study the efficient design of the aforementioned factors.

The flaxy-epoxy composite blade is compared to the glass-epoxy composite blade, using finite element methods that are developed in the early days of manufacturing methods [3]. The flaxy-epoxy composite blade outperforms the glass-epoxy composite blade in terms of safety and structural stability. According to structural and aerodynamic characteristics, the chord length and twist angle is more important on the designing of wind blades [4]. The incremental load method is used to examine the progressive failure of the composite sandwiched wind blade [5]. The wind turbine must be capable of operating in conditions of high load stress and high wind speed; the stress analysis of wind turbines need to be carried out in the wind energy conversion systems [6]. Based on discussions of blade deformation and stress, fluid–structure interaction research of wind blades was conducted [7]. The angle of attack and wind speed of the three different airfoils—S811, S822 and S826—used to produce the three and five-blade configurations, based on structural steel and aluminum alloy, were analyzed [8]. A 750 kW horizontal axis wind turbine system made of E-glass/epoxy composite material-based wind blades were designed and analyzed, where it was found that the proposed blade has a number of advantages over the ones currently in use, including safety, stability under a variety of stress conditions, and resistance to a variety of loads, including aerodynamic loads, ice build-up loads, hydrothermal loads, and mechanical loads [9]. The different wind speed predicting techniques are examined over the course of a year in order to find the best wind forecast techniques [10]. A long-term wind speed pattern, the Markov chain, the Kalman filter, the autoregressive integrated moving average, and other forecasting techniques are all investigated. The theoretical and experimental investigation of the microturbine for the low-speed zone is proposed, where the corresponding array of conventional wind turbines is used in place [11]. It was looked at if it was possible to raise the power coefficient for the relevant pitch angle and low wind velocity [12]. The performance of WECS at low wind speeds of 3 m/s to 14 m/s can be improved by using several blades on the micro wind turbine [13]. The most important component of WECS is the rotor, and the efficiency of the rotor is dependent on the airfoil and its aerodynamic design. The many options for enhancing the airfoil and its design were researched, and detailed conclusions were obtained [14]. The right airfoil was used when designing the rotor blade using the composite material of glass fiber-reinforced plastic. As a result, the average power coefficient is effectively increased to 41.2%.

In addition to creating a micro wind turbine with the appropriate wind blades, rotating the wind blade at a low wind speed is fairly challenging. The measurement of the cut-in and cut-off wind speeds was explored using a variety of estimating techniques [15]. These factor-based blade wind turbine designing approaches were presented, since the tip ratio and the torque of the rotor are the key influences on the cut-in and cut-off wind speeds. The functionality of a very compact wind turbine system has multiple advantages and disadvantages that were generates power with respect to the mode of operation [16]. The turbine’s rotor measures 500 mm in diameter. Energy output, turbine speed, power coefficient, and turbine torque were tested for a wide variety of free stream velocities. The flow surrounding the wind turbine and the effects of turbulence were examined using particle image velocimetry. The experimentally determined power coefficients were 0.4 and 0.36 under maximum and rated running conditions, respectively. An optimal driving scenario produced a tip speed ratio of 2.7. The performance of a wind turbine is superior to other commercial turbines at a slow turbine speed. Utilizing flow visualization and PIV data, the approaching flow velocity and the accelerated flow field passing the blade tip were calculated. It was found that the actual flow rate through the blades was around 20% less than the anticipated rate. Similar to compact wind turbine, the tip vortex ejected from the blade tip was also clearly visible.

When compared to conventional wind turbines, the efficiency of the small, inexpensive, and basic micro wind turbine is lower, but the cost per watt is higher. Large improvements in micro wind blade performance are theoretically possible; however, hardware implementations require additional research and hardware modifications [17].

The key component of wind turbines is how the wind blades are designed. The wind blades used in the microturbine should be feasible economically and have the lowest cut-in speed. The cut-in speed can be reduced; besides, the power coefficient and output can be increased by using more blades in the wind turbine. However, using additional wind turbine blades raises the system’s overall cost. The blade element momentum (BEM) theory codes and computational fluid dynamics (CFD) can be used to assess and investigate the performance of the micro wind turbines. In terms of faster calculation, BEM theory codes outperform CFD [18].

In order to achieve the low cut-in wind speed, a low Reynolds number airfoil is employed in the tiny horizontal axis wind turbines [19]. For enhancing the performance of tiny horizontal axis wind turbines, one root with the three primary airfoils structure has been created [20]. Compared to a traditional structure, this one has a higher lift-to-drag ratio. Using the appropriate wind tunnel tests, six airfoils, including E387, FX63-137, S822, S834, SD2030, and SH3055, were examined [21]. When the various airfoil properties were evaluated, it was discovered that the E387 airfoils performed better than the others. The outcomes of the experiment also emphasized the benefits of the other five airfoils. The deposition method for creating and printing the materials used in wind turbine design is equally crucial to airfoil designs and structural analyses. For developing and manufacturing wind blades, a material with a low deformation rate and good stress management capacity is a superior choice.

One of the manufacturing processes used to make wind turbine blades is the fused deposition modelling (FDM) technique. By using a layer-by-layer construction method, it is simple to build any complex wind blade design. The FDM method’s many steps have been explained for modelling the wind turbine [22]. Additionally, it is proposed to construct short horizontal axis wind turbine blades with FDM-assisted composite materials [23]. This method significantly improves performance. The use of 3D printing for wind turbine modelling was suggested. This designing and printing process involves many steps. The wind blade was first created as a 3D model using CAD software. Additionally, Addictive Manufacturing (AM) has integrated the blade’s 3D model into the hardware framework [24].

Development of designing software and manufacturing technologies are adding some advantages in proper designing of wind blades with improved efficiency. The QBLADE designing software is used, which improves the design capabilities [25]. This highly enhances the efficiency of blades and saves time and cost on designing. FDM-based 3D printing technology is used further for the manufacturing of the wind blade. This highly increases the roof-top power generating units. The aerodynamic testing of the 3D printing technology is carried out. From the observations, the quick designing, manufacturing, and testing of the micro wind turbines increased the efficiency [26]. The vertical axis wind turbine prototype is tested with modified geometrical parameters for the understanding the characteristics [27]. Additionally, a study has been carried out for obtaining the appropriate printing configurations, which focus on the influence of layer height on the roughness and the printing time of wind blades.

The design and production of a small-scale Gorloy helical type VAWT replica using 3D printing are proposed [28]. In comparison to other conventional wind turbines already in use, this VAWT starts generating power output with higher quality. By correctly designing wind blades with a modified proportion of infill during the 3D printing process, the performance can be further improved. With increased power generation, the infill quantity has a significant impact on the weight of the wind turbine blades. The following observations were made based on the available literature.

- The right designing, manufacturing, and printing techniques can improve the wind turbines’ performance.
- Power can be produced at low wind speeds with the right wind blade design. The material used to make wind blades can also improve their physical qualities.
- The structural characteristics also affect the wind blade’s lifespan.
- For achieving the above factors, a new kind of micro wind turbine is being designed in this paper. The major advantages of the proposed paper are as follows.
- The S9000 airfoil has been chosen for the validation of the proposed work because, according to results obtained using the Q-Blade software [2], it offers the most advantages among the ten highlighted airfoils.
- Acrylonitrile butadiene styrene (ABS) and poly lactic acid (PLA)-based wind blades were investigated and compared.
- The 100W micro wind turbine blade was created using the BEM theory method, and the CREO CAD 3.0 software was used to model the wind turbine blade in three dimensions.
- Analysis was done on Young’s modulus, density, and the Poisson ratio of wind blades made of PLA and ABS materials.
- OptiMatter software was used to obtain the infill, which ranges from 10% to 100%, and it was applied to the wind blades.
- ANSYS was then used to analyze the properties of the wind blades.

Compared to the existing wind turbines, the proposed wind turbine offers the best structural and physical properties. The proposed wind turbine blades’ density, Young’s modulus, and Poisson ratio were evaluated using the OptiMatter Tool. The proposed wind turbine has a low deformation rate and high stress and strain management rate, according to an analysis of the structural characteristics of the wind turbine in terms of deformation, stress, and strain. These characteristics give the proposed wind turbine a long lifespan and improved performance. In this work, the precise experimental details were provided together with precise waveforms and results.

## 2. Methodology

### 2.1. Modelling and Fabrication of Blade

For blade design, numerous parameters, such as power, tip speed ratio, chord, and twist, are investigated, and specifics are provided below. Micro wind turbines have quite different aerodynamic properties from large-scale wind turbines. The Reynolds number of an airfoil, which is defined by Equation (1) has a significant impact on the aerodynamic performance of a wind turbine.

(1) $$R_{e}=\frac{\rho VC}{\mu }$$

where R*_e_* is the Reynolds number, *V* is the velocity of wind (m/s). The Reynolds number is directly proportional to wind speed and chord length. These two factors had more significance on the performance of wind turbine. The wind turbine has smaller number of Reynolds number, which has less lift coefficient and high drag coefficient. The objective of the proposed work is to design a micro wind turbine with the airfoil shape with maximum drag-to-lift ratio. The wind turbine output power can be expressed as Equation (2).

(2) $$P_{r}=\frac{1}{2}C_{p}\rho V^{3}A$$

where *C_p_* is the power coefficient, *A* is the swept area of rotor. The tip ration can be defined as the ration between the speed of the rotor to the wind speed, as given in Equation (3).

(3) $$\lambda =\frac{\Omega R}{V}$$

The maximum power coefficient of the wind turbine depends on two important factors, such as chord length and twist angle. The expression for measuring the chord length and the twist angle is given in Equation (4).

(4) $$C=\frac{8\pi r\sin\alpha }{3\lambda_{r}B}$$

**Figure 1:**
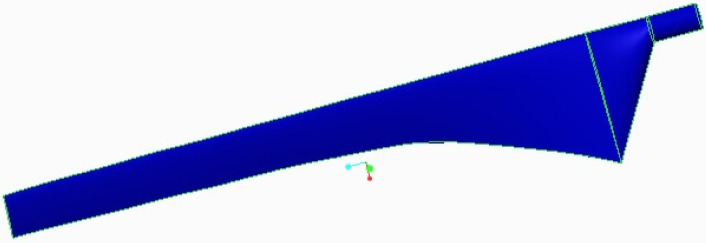
Geometric model of blade.

In this work, a new kind of wind turbine blade is designed using the BEM theory method. Almost 10 kinds of airfoils were analyzed under various categories, such as a maximum lift-to-drag ratio, and lift coefficient with distinctive angles of attack, where, the airfoil S9000 more suitable than the other airfoil, for low-speed wind applications. The three-dimensional model of the micro wind turbine is designed using the geometrical modelling tool CREO CAD 3.0. The details of the wind turbine is given in Table 1 and the geometrical model of the blade is shown in Figure 1.

**Figure 2:**
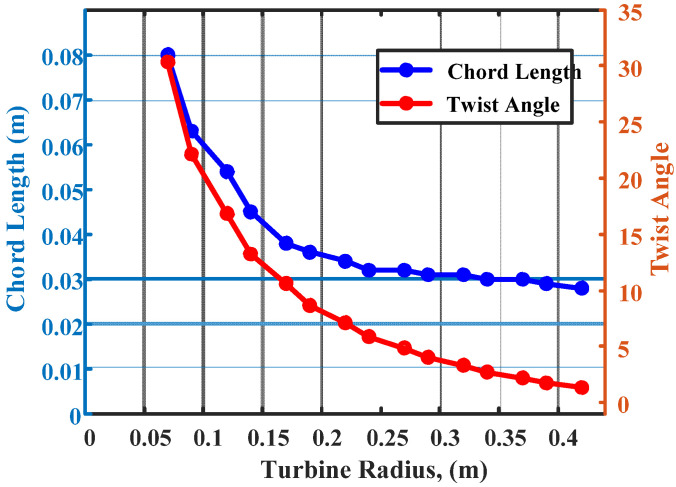
Variation of chord length and twist angle with respect to turbine radius.

The variation of chord length and twist angle of the wind blade, with respect to the turbine radius, is shown in Figure 2. The variation of twist angle and chord length, with respect to the turbine radius, is given in Table 2. The chord length is the length between the distance from the front edge of the wind blade to the back edge of the wind blade. Chord length can make an impact on the performance of wind blades. The efficient wind blades must have a linear chord width and reduced twist angle. The relationship between the radius of the wind turbine and twist angle and chord length is given in Table 2. The air inflow angle, which lifts the blades when rotating, is the twist angle. The twist angle is highly dependent on the rotational speed, the velocity of wind speed, and the radius of the turbine. The radius of the proposed wind turbine is 0.42 m. When the radius of the wind turbine is increased, the chord length and the twist angle get reduced, as shown in Figure 2. At a radius of 0.12 m, the proposed wind turbine has a twisting angle of 17 degrees and a chord length of 0.05. It gets reduced with the increase in the radius dimension. At a radius of 0.42 m, the proposed turbine generates effective power at the low wind speed.

The capability of temperature maintenance in the wind turbine is an important factor in the modelling. The high temperature in the turbine and blade will reduce the air density, whereas the low temperature will freeze the blades. According to research from the United States Environmental Protection Agency (EPA), the average life span of a wind turbine is roughly 20 years. Using 3D printing technology for the manufacture of thermoplastic blades may significantly improve the recyclability of the blades. The 3D printed technology for the wind blades needs the head bed print for maintaining the low bed temperature. However, in the proposed work the wind blade is composed of the PLA material, which does not require any additional head printing. The heat-printed beds are quite costly, whereas the PLA material avoids the cost of the above. The use of ABS and PLA with 3D printing in the wind turbine is reducing the cost.

### 2.2. Wind Tunnel Setup

**Figure 3:**
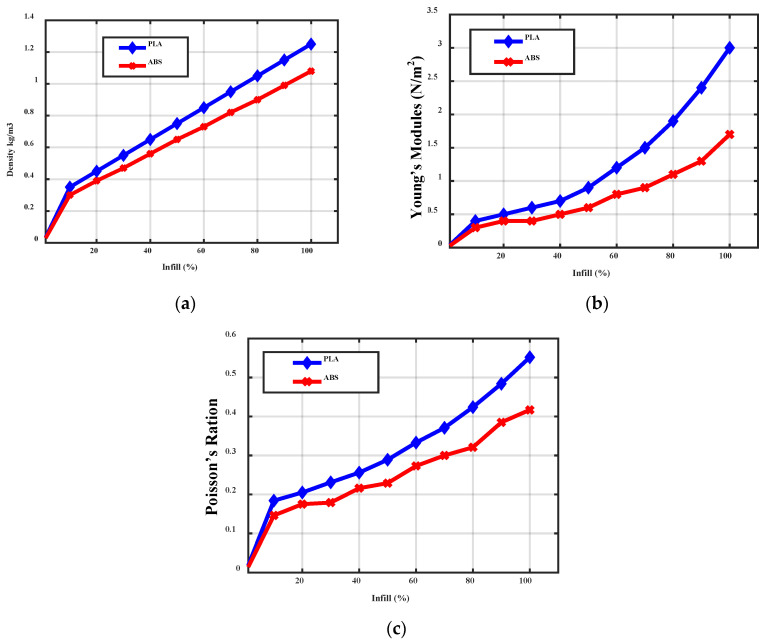
Comparison of (**a**) Blade density (**b**) Young’s modulus (**c**) Poisson ratio with respect to infill percentage of PLA and ABS material.

The experimental setup of the proposed wind turbine is shown in Figure 3. The diameter of the wind tunnel exit is 109 cm for the proposed work. A three-phase AC motor of the centrifugal fan with a rotational speed of 1300 rpm and rated power of 5 Hp is used for the wind tunnel motor. The wind turbine blades are attached to the hub with the permanent magnet generator. The above setup is kept in front of the nozzle. For measuring the wind speed through the wind tunnel, the Lutron AM-4201 model digital anemometer with a range of 0.4 m/s to 30 m/s, resolution of 0.1 m/s, and with the accuracy of (±2% + 0.1 m/s) is used in the experimental setup. An LCD digital multi meter of Lutron DT830D has been used to measure the generated voltage and current from the wind turbine. Two wipro Garnet base B22-50W LED bulbs were used as the load for the wind turbine. The 5-bladed proposed wind turbine model with the airfoilS9000 with the above-discussed equipment was arranged together for analyzing the performance at different wind speeds.

Wind turbine rotor speed is measured by a hand-held laser tachometer at the rotor and tip of the blade. The wind velocities are measured at the outlet of the wind tunnel using a digital anemometer. Wind velocity has been controlled inside the wind tunnel for experimentation. At the wind speed of 3 m/s to 8 m/s, the proposed wind turbine efficiently generated power. When the wind speed was raised beyond 13 m/s, the wind turbine experienced vibration due to an aeroelastic phenomenon. Therefore, the wind turbine was not evaluated in excess of 12 m/s. The wind turbine blade tip velocity ratio was discovered to rise with increased wind speed between 7 and 8 m/s.

## 3. Results and Discussion

### 3.1. Properties of Blade Material

OptiMatter is a provider of a cloud-based 3D printing materials database. It provides data on material performance, quality, and processability. Its features include material selection for 3D printing, as well as the discovery of optimal printing parameters. Based on the requirement, the OptiMatter can provide the solution. It completely reduces the time and cost of improving the outcome of the prints. Infill pattern is the structure and shape of the material inside of a part. Ranging from simple lines to more complex geometric shapes, infill patterns can affect a part’s strength, weight, print time, and even flexibility. The proposed wind blades of PLA and ABS materials are printed with the infills, with different ratios of 10% to 100% is analyzed in this section. The material properties, such as density, Young’s modulus, and the Poisson ratio, with respect to the infill ratio were analyzed.

Generally, the material used for the wind turbine blades should be low density for efficient durability. The density of the proposed wind turbine blades is analyzed with respect to the infill from 10% to 100%. The PLA material has 0.35 kg/m^3^ on the 10% of infill on the material, whereas the ABS material has a density of 0.3 kg/m^3^. At 50% of infill, the PLA and ABS material-based wind turbine blade has a density of 0.75 kg/m^3^ and 0.6 kg/m^3^, respectively. On 100% of infill, the PLA and ABS have a density of 1.25 kg/m^3^ and 1.08 kg/m^3^, respectively. The PLA and ABS materials have similar values of density, as compared to the existing composite materials. The physical properties comparison of PLA and ABS materials-based wind blade are given in Table 3. 

Young’s modulus is a property of the material that defines the elasticity and stretchability in tension or compression. It is a physical property of material, which defines its manageable stress and strain of it. The Poisson’s ratio is the ratio of change in the width to the change in length under the corresponding strain applied to it.

Figure 3 shows it is evident that the density, Young’s modulus, and Poisson’s ratio of the PLA material are higher than the ABS material for the same infill percentage. Further, the data obtained from OptiMatter is considered for structural analysis through ANSYS 15.

### 3.2. Structural Analysis of Blade

The force in the wind acts as the axial load, which creates stress, deformation, and strain, on the wind blades. The axial load acts on the wind blade are measured by the following parameters, such as wind velocity, blade length, and cross-sectional area. The structural analysis of the proposed micro wind turbine with both the PLA and ABS material has been analyzed in terms of total deformation, equivalent stress, and equivalent strain. The above structural analysis is done using the ANSYS 15 tool. The above characteristics of the wind turbine are analyzed with the infill range of 10% to 100%. 

**Figure 4:**
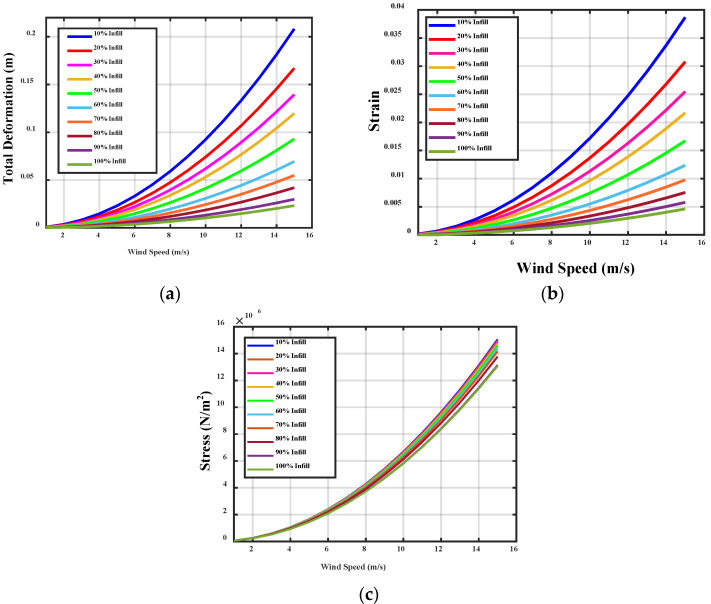
Variation of (**a**) total deformation (**b**) equivalent strain (**c**) equivalent stress, with respect to wind velocity for different ratios of PLA at 0.3 layer heights.

**Figure 5:**
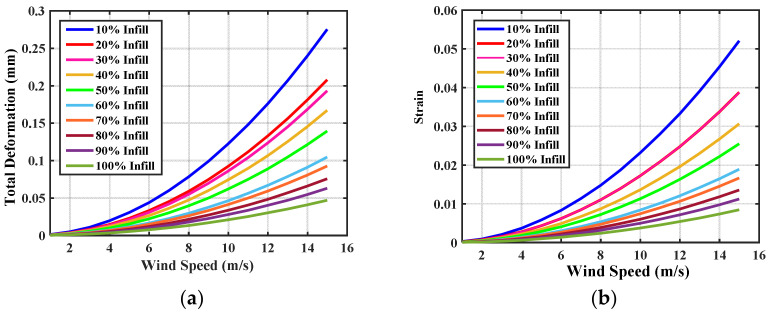

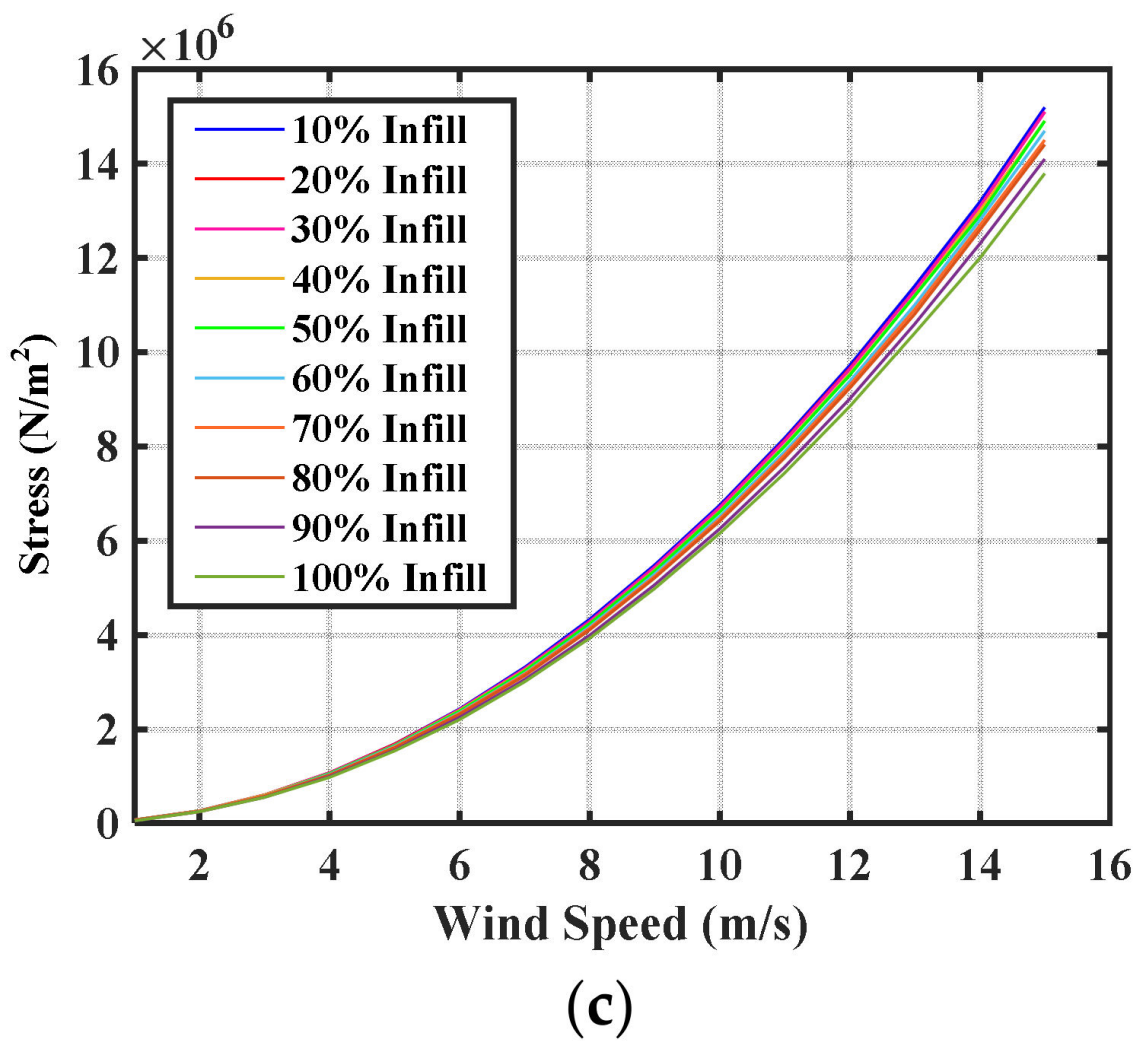
Variation of (**a**) total deformation (**b**) equivalent strain (**c**) equivalent stress, with respect to wind velocity for different ratios of ABS at 0.3 layer heights.

The deformation of material is the change in physical property due to the stretching, squeezing, and twisting while applying an external force. The wind turbine blade is exposed in the open environment and experiences too much force during the attack of wind. The wind blade is stricken by the wind and it starts to rotate. The air force causes stress on the wind blade. The efficient wind turbine blade must be capable to resist deformation, stress, and strain during the external applying force. The proposed PLA material-based wind turbine blade and ABS material-based wind turbine blade were analyzed using the ANSYS 15. An infill of 0.29 mm layer height is applied on the wind blade, which also made an impact on the structural properties. The deformation, stress, and strain were analyzed for the PLA and ABS material-based wind turbine blade with infill of 10% to 100%. The variation of total deformation, equivalent strain, and equivalent stress, with respect to wind velocity for different ratios of PLA and ABS Material at 0.3 layer heights, is observed and plotted, as shown in Figure 4 and Figure 5.

**Figure 6:**
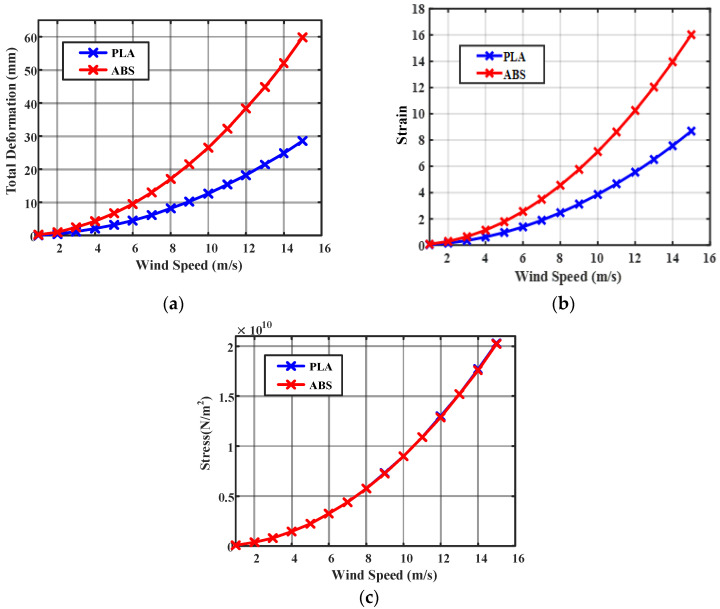
Comparison of PLA and ABS material (**a**) total deformation (**b**) strain (**c**) stress, with respect to wind speed (0.3-layer heights at 90% Infill).

A comparison has been carried out for measuring the total deformation, strain, and stress, with respect to the wind speed for both PLA and ABS material with 90% of infill and 0.28 mm layer height is shown in Figure 6. The structural properties of the PLA and ABS materials based wind blades are given in Table 4. 

**Figure 7:**
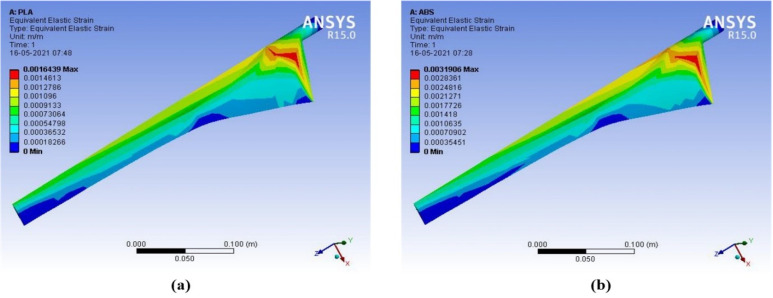
Contour of equivalent strain (**a**) ABS (**b**) PLA (wind velocity = 8 m/s).

**Figure 8:**
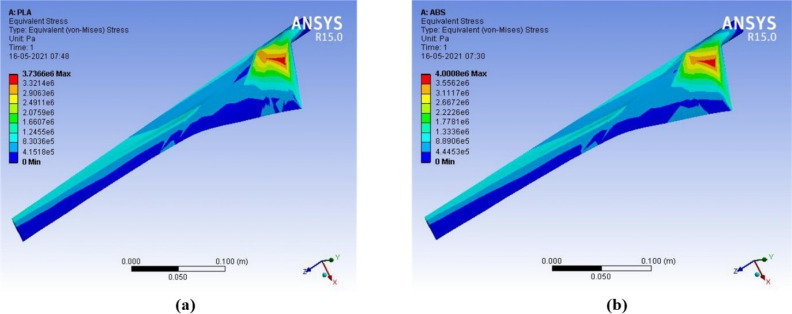
Contour of equivalent stress (**a**) ABS (**b**) PLA (wind velocity = 8 m/s).

**Figure 9:**
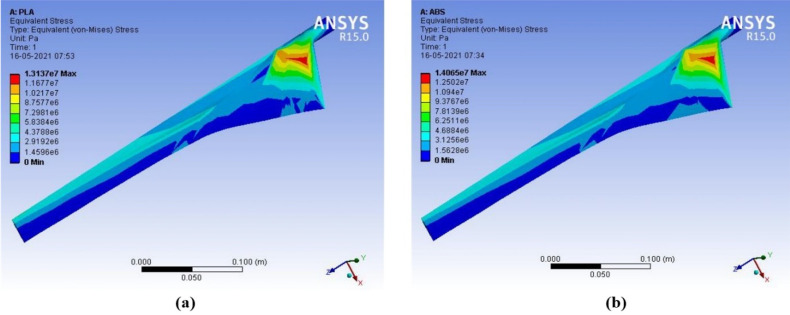
Contour of equivalent stress (**a**) ABS (**b**) PLA (wind velocity = 15 m/s).

**Figure 10:**
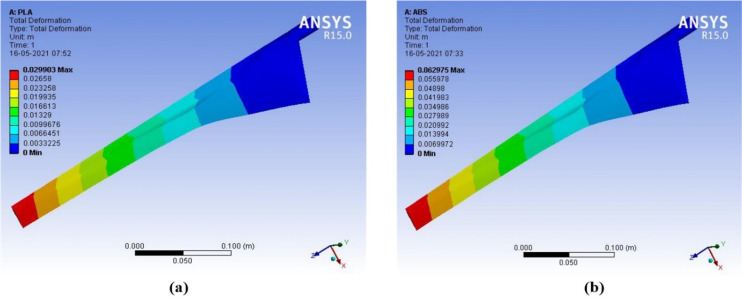
Contour of total deformation (**a**) ABS (**b**) PLA (wind velocity = 15 m/s).

The output results of deformation, equivalent stress, and equivalent strain are compared for ABS with PLA materials. When wind speed increases between 4–15 m/s, the deformation in PLA material is 2.13 × 10^−3^ m to 29.90 × 10^−3^ m and the deformation in ABS material is 4.48 × 10^−3^ m to 62.98 × 10^−3^ m. PLA material has less deformation than ABS material at different wind speeds. PLA material has better structural characteristics than ABS material. PLA is a powerful, highly durable, and chemically resistant thermoplastic by itself. Fine and smooth finishing to the blades was given by filling on the surfaces. The force in the wind acts as the axial load, which creates stress, deformation, and strain on the wind blades. The axial load acts on the wind blade are measured by the following parameters, such as wind velocity, blade length, and cross-sectional area. The structural analysis of the proposed micro wind turbine with both the PLA and ABS material has been analyzed in terms of total deformation, equivalent stress, and equivalent strain. The above structural analysis is done using the ANSYS 15 tool. The above characteristics of the wind turbine are analyzed with an infill range of 10% to 100%. The CFD analysis for the proposed wind blade has been performed in the ANSYS. This analysis allows the domain to create the constraints, such as inlet, outlet, amount of pressure, and the thickness of the wind tunnel. Based on the measured pressure, the aerodynamic forces acted on the blade surface can be obtained. This analysis helps to determine the equivalent stress and strain created on the blade surface which defines the structural characteristics of the wind turbine. The contour of strain, stress, and deformation has been taken through CFD analysis using the ANSYS 15 software, as shown in Figure 7, Figure 8, Figure 9 and Figure 10, respectively.

### 3.3. Performance Analysis

**Figure 11:**
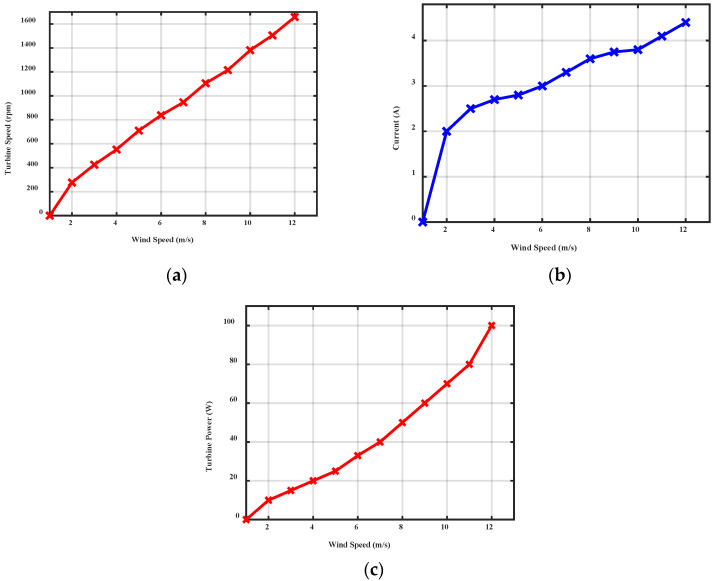
Variation of (**a**) turbine speed (**b**) current (**c**) turbine power, with respect to wind velocity.

The enhanced performance of the proposed micro wind turbine rotor blade with the length of 0.41 m is analyzed. From the structural analysis, the airfoil S9000 is used for the experimental investigations. The performance of the proposed wind turbine is initially influenced by the chord, twist distribution from root to top, tip speed ratio, and aerodynamic performance of the airfoil. The S9000 airfoil generates maximum power of 100 watts at the wind speed of 12 m/s. The current generation from the wind turbine is increased, with respect to the wind speed. Additionally, the rotor angular speed is linearly increased, with respect to the wind speed. The value of current and voltage generation is measured using the two multimeters, whereas the wind flow has linearly increased from 2 m/s to 12 m/s. The performance of the proposed wind model, with respect to the wind velocity is shown in Figure 11.

The wind turbine starts to produce power output at the cut in wind speed of 2 m/s. At the 2 m/s of wind speed, the wind turbine generates 10 W of power output, whereas it linearly increases, with respect to the wind speed. At a wind speed of 8 m/s, the turbine generates the output power of 50 W. The turbine generates the rated power output of 100 watts at the wind speed of 12 m/s. 

The PLA material base wind turbine has started to generate power at low wind speed. As compared with the conventional wind turbines, the proposed micro wind turbine has generated more power with better structural properties. The structural properties, in terms of deformation rate and withstand capability of stress and strain have been analyzed with the necessary validations. The proposed micro wind turbine has the best structural properties of the conventional wind turbines.

## 4. Conclusions

In this paper, a new kind of efficient 100W micro wind turbine was proposed. The proposed wind turbine was constructed as a horizontal axis wind turbine with the PLA material. The CREO CAD 3.0 software was used for the designing of the wind blade’s 3D CAD model. Based on this model and with the appropriate 3D printing fusion deposition method, the proposed 100 watts micro horizontal axis wind turbine was fabricated. Further, 0.29 mm layer height of infill was applied on the PLA and ABS material-based wind turbines using the OptiMatter. The properties of the wind turbine blade were analyzed in terms of density, Young’s modulus, and Poisson ratio. Then, the structural analysis of the PLA and ABS materials-based wind blades was performed using the ANSYS 15 tool. In structural analysis, the deformation, stress, and strain experienced by the wind blade during the axial load were examined. The performance of wind turbines at the low wind speed to rated speed was analyzed in terms of turbine speed, the current generation, power output, deformation rate, stress, and strain. In conclusion, the PLA material-based wind turbine is the best for the wind energy conversion system because of its low deformation rate, experiencing less stress and less strain during the axial load. From the detailed experimental analysis and with the corresponding results, the proposed PLA material-based wind turbine can generate maximum power output at a low wind speed and have a better performance than the other existing wind turbines.

## Figures and Tables

**Table 1:** Details of wind turbine.

S. No	Parameters	Range
1	Power	100 W
2	Profile	S9000 (9.2%)
3	Axis of rotation	Horizontal
4	No of Blades	5
5	Radius of the Blade	0.41 m
6	Root Chord Length	0.0802 m
7	Tip Chord Length	0.0280 m

**Table 2:** Chord and twist distribution of various sections.

S. No	Turbine Radius (m)	Twist Angle (^O^)	Chord Length (m)
1	0.07	29	0.072
2	0.09	21	0.057
3	0.11	16	0.049
4	0.13	12	0.041
5	0.16	10	0.034
6	0.18	9	0.032
7	0.21	7	0.031
8	0.23	6	0.029
9	0.26	5	0.029
10	0.28	4	0.028
11	0.30	3	0.028
12	0.32	3	0.027
13	0.35	2	0.027
14	0.37	2	0.026
15	0.40	1	0.025

**Table 3:** Physical properties comparison of PLA and ABS materials-based wind blade.

S. No	Percentage of Infill	PLA			ABS		
		Density kg/m^3^	Young’s Modulus (N/m^2^)	Poisson Ratio	Density kg/m^3^	Young’s Modulus (N/m^2^)	Poisson Ratio
1	0	0	0	0	0	0	0
2	10	0.32	0.36	0.17	0.27	0.27	0.13
3	20	0.41	0.45	0.18	0.35	0.36	0.16
4	30	0.50	0.54	0.21	0.42	0.36	0.16
5	40	0.59	0.63	0.23	0.50	0.45	0.19
6	50	0.68	0.81	0.26	0.59	0.54	0.21
7	60	0.77	1.08	0.30	0.66	0.72	0.25
8	70	0.86	1.35	0.33	0.74	0.81	0.27
9	80	0.95	1.71	0.38	0.81	0.99	0.29
10	90	1.04	2.16	0.44	0.89	1.17	0.35
11	100	1.13	2.70	0.50	0.97	1.53	0.38

**Table 4:** Structural properties comparison of PLA and ABS materials-based wind blade.

S. No	Wind Speed (m/s)	PLA			ABS		
		Total Deformation (mm)	Stress (N/m^2^)	Strain	Total Deformation (mm)	Stress (N/m^2^)	Strain
1	1	0.114	0.035	8.11 × 10^7^	0.239	0.064	8.07 × 10^7^
2	2	0.456	0.139	3.24 × 10^8^	0.957	0.256	3.23 × 10^8^
3	3	1.027	0.313	7.29 × 10^8^	2.153	0.577	7.27 × 10^8^
4	4	1.826	0.556	1.30 × 10^9^	3.828	1.025	1.30 × 10^9^
5	5	2.853	0.868	2.03 × 10^9^	5.982	1.602	2.02 × 10^9^
6	6	4.108	1.251	2.92 × 10^9^	8.614	2.307	2.91 × 10^9^
7	7	5.592	1.702	3.97 × 10^9^	11.724	3.141	3.96 × 10^9^
8	8	7.303	2.223	5.18 × 10^9^	15.313	4.102	5.17 × 10^9^
9	9	9.243	2.814	6.56 × 10^9^	19.381	5.191	6.54 × 10^9^
10	10	11.411	3.474	8.11 × 10^9^	23.927	6.409	8.07 × 10^9^
11	11	13.808	4.203	9.81 × 10^9^	28.951	7.755	9.81 × 10^9^
12	12	16.432	5.002	1.17 × 10^10^	34.454	9.230	1.16 × 10^10^
13	13	19.285	5.871	1.37 × 10^10^	40.436	10.832	1.37 × 10^10^
14	14	22.367	6.809	1.59 × 10^10^	46.896	12.562	1.58 × 10^10^
15	15	28.529	8.6845	2.03 × 10^10^	59.816	16.023	2.02 × 10^10^

## Data Availability

The data presented in this study are available on request from the corresponding author.
